# Recent Advances in Oxygen Electrocatalysts Based on Perovskite Oxides

**DOI:** 10.3390/nano9081161

**Published:** 2019-08-14

**Authors:** Jun Xu, Chan Chen, Zhifei Han, Yuanyuan Yang, Junsheng Li, Qibo Deng

**Affiliations:** 1 Department of Chemistry, School of Chemistry, Chemical Engineering and Life Sciences, Wuhan University of Technology, 122 Luoshi Road, Wuhan 430070, China; 2 School of Materials Science and Engineering, Tianjin University of Technology, Tianjin 300384, China; 3 Hubei Provincial Key Laboratory of Fuel Cell, Wuhan University of Technology, 122 Luoshi Road, Wuhan 430070, China

**Keywords:** perovskite oxide, oxygen reduction reaction, oxygen evolution reaction, electrocatalyst

## Abstract

Electrochemical oxygen reduction and oxygen evolution are two key processes that limit the efficiency of important energy conversion devices such as metal–air battery and electrolysis. Perovskite oxides are receiving discernable attention as potential bifunctional oxygen electrocatalysts to replace precious metals because of their low cost, good activity, and versatility. In this review, we provide a brief summary on the fundamentals of perovskite oxygen electrocatalysts and a detailed discussion on emerging high-performance oxygen electrocatalysts based on perovskite, which include perovskite with a controlled composition, perovskite with high surface area, and perovskite composites. Challenges and outlooks in the further development of perovskite oxygen electrocatalysts are also presented.

## 1. Introduction

Electrochemical oxygen reduction reaction (ORR) and oxygen evolution reaction (OER) are the key processes in a variety of energy conversion devices such as fuel cells and metal–air batteries [1,2]. Both ORR and OER have high activation barriers that severely limit the overall performance of the energy conversion devices in which ORR/OER are involved. Noble metal-based electrocatalysts have long been used to catalyze ORR (e.g., Pt) and OER (e.g., IrO_2_ and RuO_2_). However, noble metal electrocatalysts suffer from several inherent limitations. First, precious metal electrocatalysts are susceptible to poisons and tend to aggregate under working conditions, leading to the degradation of performance [3,4]. Furthermore, the low availability of noble metals cannot sustain their widespread applications as an electrocatalyst in emerging energy conversion devices [5]. In addition, electrocatalysts are required to have high catalytic activity toward both ORR and OER in some specific applications such as a metal–air battery [6]. The bifunctionality of the noble metal electrocatalysts is not high enough to meet the demand of these devices. Thus, exploration of low-cost and high-performance oxygen electrocatalysts has gained significant research interest recently. Carbon-based electrocatalysts that feature good conductivity, large surface area, and tunable heteroatom content are now shown to be promising oxygen electrocatalysts [5,7,8]. For example, a N and S co-doped carbon catalyst demonstrated a superior ORR and OER activity that surpassed most of the existing oxygen electrocatalysts [9]. However, the oxidation of carbon materials at anodic conditions leads to the generation of oxygen species on the surface, which will increase the resistance and may eventually give rise to the decomposition of the materials [10,11]. Thus, the long-term stability of carbon-based materials in a highly oxidative OER environment is a great challenge.

An ideal oxygen electrocatalyst should offer proper binding (neither too strong nor too weak) to oxygen species. Transition metal oxides are a class of low-cost material that could provide d orbital for the binding of oxygen species. Thus, transition metal oxides are considered to be a viable alternative to the traditional precious metal oxygen electrocatalysts. Perovskite oxides with the general formula ABO_3_ are generally defined as oxides that have the same crystal structure with CaTiO_3_. Normally, the large-radius rare-earth ions occupy the A-site that are 12-fold coordinated with oxygen ions, while the B-site is occupied by 6-fold coordinated transition metal ions. A trait of perovskites that differ from other metal oxides such as spinels is their capability of accommodating multiple A-site and B-site cations. This provides an opportunity to finely tune its electronic and catalytic properties [12]. In addition, perovskite oxides allow for partial substitution at the A and/or B position [13,14]. This partial substitution can induce changes in the valence states of A- and B-site cations and the generation of oxygen vacancies. These changes in perovskite oxides can be used to engineer the adsorption behavior of reaction intermediate on the material, and thus their catalytic activity can be improved. Furthermore, perovskite oxides are chemically more stable than the carbon-based catalysts under oxidative electrochemical reactions. Although reports on perovskite oxygen electrocatalysts can be dated back to the 1970s [15], perovskites have only recently been recognized as a sort of competitive oxygen electrocatalysts [16,17], which is possibly due to the lack of understanding on the mechanism of the oxygen electrocatalysis. In this review, we aim to cover the advances in active perovskite oxygen electrocatalysts that have recently been reported. The first part of the review focuses on intrinsic ORR/OER activity of perovskite oxides. Subsequently, we discuss emerging perovskite oxygen electrocatalysts that show high ORR/OER activity. Finally, a brief summary and outlook for the future development of advanced perovskite electrocatalysts is provided.

## 2. Intrinsic ORR/OER Activity of Perovskite Oxides

**Figure 1:**
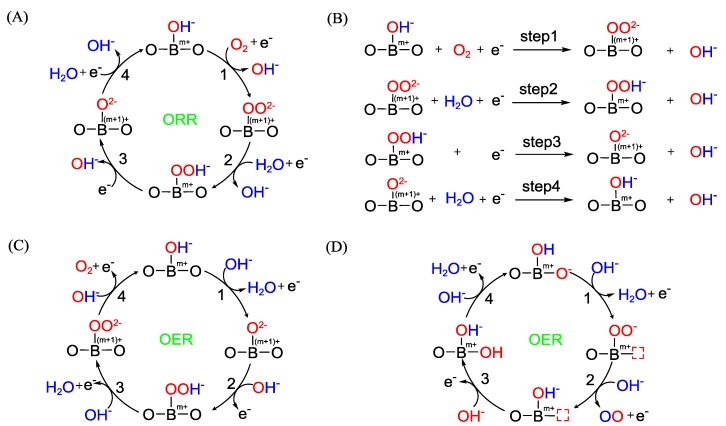
Proposed (**A**) Oxygen reduction reaction (ORR) mechanism (reproduced from [16], with permission from springer Nature, 2011 ) for perovskite electrocatalysts. (**B**) the detailed steps of ORR by equations. (**C**, **D**) Two different Oxygen evolution reaction (OER) mechanisms proposed for perovskite electrocatalysts (reproduced from [26]).

The catalytic mechanism of perovskite oxides is distinct from that of a precious metal catalyst and its ORR/OER activity is mainly attributed to the transition metal site [18,19,20]. A generally-accepted ORR pathway on a perovskite oxides is shown in Figure 1A,B [16,21]. Briefly, the oxygen atom of an H_2_O molecule is coordinated to the transition metal on the surface, generating surface M-OH^−^ species. The dissociative adsorption of the oxygen molecule, through either a side-on or an end-on fashion, leads to the oxidation of M and displacement of surface OH^−^ to O_2_^2−^ (Step 1). Surface O_2_^2−^ is protonated with M being reduced (Step 2), followed by the oxidation of M and formation of surface O^2−^ (Step 3). Subsequent O^2−^/OH^−^ displacement results in the regeneration of surface hydroxide. Step 1 and 4 are thought to be the rate-determining steps. According to this mechanism, the binding strength of oxygen on the perovskite surface influences its ORR performance. It has been pointed out that for a perovskite oxide without structural deformation or distortion, its ORR activity can be correlated with the extent of the overlap between the e_g_ orbital of the perovskite and the sp_σ_ orbital of oxygen, and perovskite with a higher overlap integral are generally more active toward ORR [22]. Suntivich et al. examined a series of perovskites in terms of their electronic structure and ORR performance and concluded that one of the main factors determining the ORR activity of the perovskites was the extent of antibonding orbital filling of the transition metal ions [16]. Perovskites with a higher e_g_ filling hinder the activation of oxygen and the binding strength of the reaction intermediate while ones with a low e_g_ filling inhibit the desorption of the ORR intermediate. An e_g_ filling of ~1 was proposed as the optimal electronic structure for ORR and this criterion was used to guide the rational design of active perovskite ORR catalysts. Although this performance descriptor has been demonstrated in many cases [23,24], e_g_ cannot be used solely to predict the ORR activity of the perovskites as it was found out experimentally that some perovskites with identical e_g_ possessed different intrinsic ORR performance [25], which suggests that the bulk electronic status of the perovskites is not an exclusive factor that determines its surface chemical activity. Other factors such as the covalency of the B–O bond [16] also affect the intrinsic ORR activity of perovskite oxides. 

Likewise, the B-site is also thought to be the main active site of OER and OER is believed to proceed on perovskite oxides in a reverse process with ORR (Figure 1C) [17,27]. Since the e_g_ orbitals of the transition metal ions in perovskite oxides could strongly overlap with the surface oxygen adsorbates [28], the e_g_ filling of a transition metal ion was also considered to be an ideal parameter to describe the intrinsic activity of a perovskite oxide. An e_g_ occupancy of ~1 was identified as optimal for a perovskite oxide OER catalyst [17]. It was found that lattice oxygen in perovskite oxides could also participate in the OER reaction and a new mechanism was suggested recently (Figure 1D) [26,29]. This mechanism is based on the observation that the Co 3d band hybridizes with the lattice O 2p π band at an applied potential, which endows the lattice oxygen with OER activity [30]. 

It should be noted that the structure and intrinsic OER activity of perovskite oxides may evolve with the OER process due to the high potential applied during OER [31,32,33]. For example, BaNiO_3_ showed limited initial OER activity. As OER proceeded, a Ni vacancy was generated and a mixed valence of Ni was formed, leading to the formation of BaNi_0.83_O_2.5_. The calculated O p-band center of BaNi_0.83_O_2.5_ was −1.79 eV, closer to the Fermi level when compared to that of BaNiO_3_. In addition, BaNi_0.83_O_2.5_ had an optimized e_g_ filling of 1.4. The surface area normalized activity of BaNi_0.83_O_2.5_ was much higher than that of BaNiO_3_ [32]. Similarly, Ba^2+^ and Sr^2+^ in Ba_0.5_Sr_0.5_Co_0.8_Fe_0.2_O_3−δ_ (BSCF5582) [17] may also leach out during OER cycles because of the redox reaction between oxygen ions and metal cations caused by the high O *p*-center relative to the Fermi level [34,35]. The leaching out of cations led to the formation of an amorphous surface layer on BSCF, thus decreasing its activity [36]. 

## 3. Active Perovskite Oxygen Electrocatalysts

### 3.1. Perovskite Oxygen Electrocatalysts with Regulated Composition

Varying the composition of perovskite oxides can adjust their electronic state and crystal structure, thereby affecting their catalytic activity toward ORR and OER. The effect of A-site and B-site cation on the ORR/OER performance of ABO_3_ type perovskites has been thoroughly investigated and reviewed elsewhere [37,38,39,40,41]. These previous studies suggest the possibility of enhancing the performance of a perovskite electrocatalyst through substitution of its A and/or B-site. In this sub-section, we focus on recent studies on perovskite oxides with partial cation substitution or oxygen vacancies.

#### 3.1.1. Perovskite Electrocatalysts with Partial A-Site Variation

**Figure 2:**
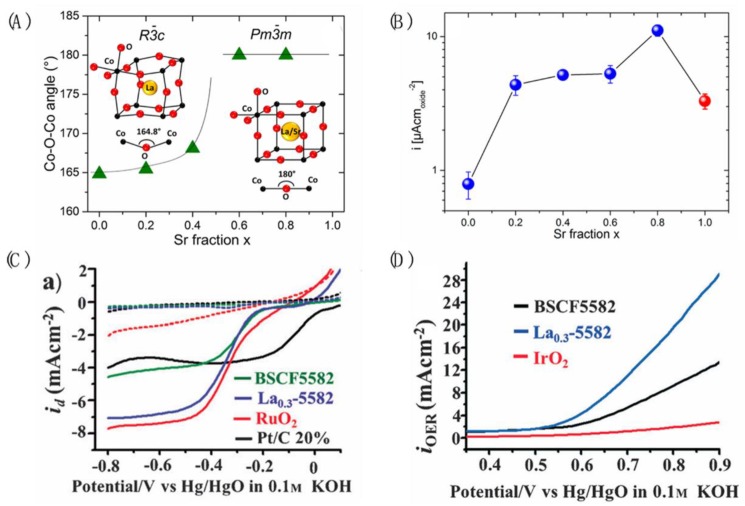
(**A**) The Co–O–Co angle in La_1−x_Sr_x_CoO_3_ as a function of Sr content (adapted from [43], with permission from American Chemical Society, 2015). (**B**) OER current density (in μA cm_oxide_^−2^) of the La_1−x_Sr_x_CoO_3_ series; the red circle represents SrCoO_2.5_ (adapted from [43], with permission from American Chemical Society, 2015). (**C**) ORR and (**D**) OER performance of KB supported La_0.3_(Ba_0.3_Sr_0.5_)_0.7_Co_0.8_Fe_0.2_O_3−δ_ in 0.1 M KOH solution at 1600 rpm with a scan rate of 10 mV s^−1^ (adapted from [23], with permission from John Wiley and Sons, 2014).

The perovskite A-site is generally not considered to be the active site that directly participates in the oxygen electrode reactions. However, A-site cations may influence the ORR/OER performance of the perovskite indirectly. It has been demonstrated that the introduction of Sr into LaCoO_3_ influences the crystal structure of the perovskite so that the atoms tend to align along the Co–O–Co bond (Figure 2A) [42,43]. Such a configuration in turn causes a higher overlap between the occupied O 2p valence band and unoccupied Co 3d band, which increases the intrinsic activity of the perovskite. In addition, incorporation of the Sr component in the perovskite also leads to the generation of Co with a higher oxidation state. Due to these effects, the Sr-doped LaCoO_3_ catalyst showed improved catalytic performance than LaCoO_3_ (Figure 2B). Similarly, the doping of La into the A-site of BSCF5582 was reported to selectively pose local stress on the Co sublattice octahedron [23]. The comminution of La_0.3_-BSCF5582 was observed due to the presence of the local stress. In addition, a secondary phase, LaCoO_3−δ_, was also generated. Furthermore, Co and Fe were partly oxidized because of the introduction of a higher valence La. These effects all together endowed La_0.3_-BSCF5582 with high ORR activity and superior OER activity, which was 2–3 times higher than the well-known BSCF5582 (Figure 2C,D). Na-doped SrRuO_3_ improved oxygen evolution activity and durability in acid media [44]. SrRuO_3_ bound reaction intermediates too strongly while Na^+^ substituted in the Sr^2+^ position not only stabilized the structure, but also increased the oxidation state of Ru so that it could positively shift the position of the O p-band and Ru d-band centers, impairing the bonds between Ru and oxygen species, which showed high OER activity and durability.

Partial substitution of A-sites in BaCoO_3−δ_ with lanthanides (Pr, Sm, Gd, Ho, and Ln) have been shown to enhance the activity and the durability of the perovskites [45]. The e_g_ filling of the substituted perovskite, Ln_0.5_Ba_0.5_CoO_3−δ_, was estimated to be ~1, which might explain its superior OER activity. DFT studies revealed that the O *p*-band center of Ln_0.5_Ba_0.5_CoO_3−δ_ was close to the Fermi level, which is important for the high activity and stability of the catalyst. In another study, a partial substitution of Ba in PrBaCo_2_O_5+δ_ with Sr was proven to modulate the surface Co^4+^ concentration [46]. The electrophilic surface Co^4+^ facilitates the formation of surface O–OH and the deprotonation of surface adsorbed OOH species [47], thereby improving the OER activity of the perovskite. The ORR activity of Sr-doped PrBaCo_2_O_5+δ_ was also enhanced due to the improved O_2_ adsorption on the oxide surface with high concentration Co^4+^. 

While substitution of A-site elements with a higher valence ion is an effective approach to enhance its electrochemical performance, a high degree of A-site substitution may cause the breakdown of the perovskite structure. Gobaille-Shaw et al. synthesized La_1−x_Ba_x_MnO_3_ nanoparticles through a highly versatile ionic-liquid based method. The ORR activity showed a significant dependence on the bulk Ba content and the best catalytic performance was obtained for x = 0 and 0.15 [48]. To obtain the stable perovskite oxides with the optimal electronic configuration, the substitution process was coupled with a hydrogenation treatment [49]. Yb-doped CaMnO_3_ (Ca_0.9_Yb_0.1_MnO_3_, CYM) were treated in a H_2_/Ar atmosphere at different temperatures from 320 °C to 400 °C. CYM hydrogenated at 350 °C had OER activity, which was judged by the OER current density at an overpotential of 500 mV, about 100 times higher than that of the pristine CYM, demonstrating the efficacy of the hydrogenation treatment. The excellent OER performance of the hydrogenated CYM was attributed to its appropriated e_g_ filling and high electron conductivity.

#### 3.1.2. Perovskite Electrocatalysts with Partial B-Site Variation

**Figure 3:**
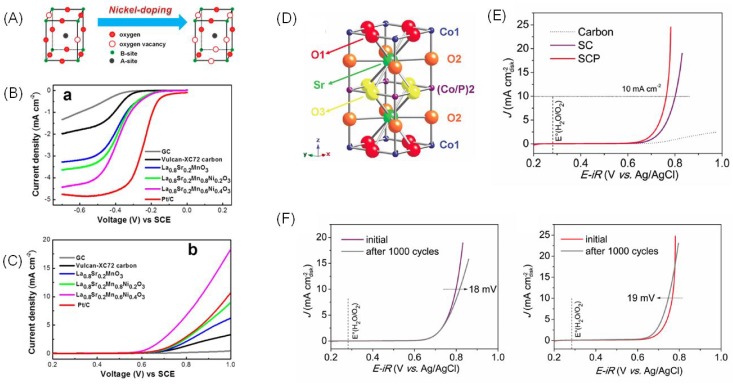
(**A**) Scheme showing the Ni doping on La_0.8_Sr_0.2_MnO_3_ (adapted from [56], with permission from American Chemical Society, 2016). (**B**) ORR and (**C**) OER performance of La_0.8_Sr_0.2_Mn_0.6_Ni_0.4_O_3_ in O_2_-saturated 0.1 M KOH (adapted from [56], with permission from American Chemical Society, 2016) (**D**) Scheme showing the tetragonal crystal structure of SrCo_0.95_P_0.05_O_3−δ_ (SCP) (adapted from [60], with permission from John Wiley and Sons, 2016). (**E**) OER performance of SCP and SrCoO_3−δ_ (SC) in O_2_ saturated 0.1 M KOH solution with a scan rate of 5 mV s^−1^ at 1600 rpm (adapted from [60], with permission from John Wiley and Sons, 2016). (**F**) OER LSV curves of CP (left) and SCP (right) before and after accelerating degradation tests (adapted from [60], with permission from John Wiley and Sons, 2016).

Since B-site directly participates in oxygen redox reactions, the B-site replacement is a simple and effective way to control the ORR/OER activity of a perovskite catalyst [50,51,52]. Through partial substitution of B-site in SrTiO_3−δ_ with the transitional metal element (M = Co or Fe), the active perovskite catalyst can be obtained [53]. When Co was used as the B-site element, the OER activity of the substituted SrCo_0.9_Ti_0.1_O_3−δ_ approached that of BSCF. In addition, a better durability was demonstrated for SrCo_0.9_Ti_0.1_O_3−δ_. The good OER performance of SrM_0.9_Ti_0.1_O_3−δ_ stems from its optimized e_g_ filling (~1.16). Furthermore, the low Co–O bond strengthens, which facilitates the formation of the redox-active oxygen vacancy and could also contribute to its high activity. Subiao Liu et al. first reported Co-doping layered perovskite LaSr_3_Co_m_Fe_3−m_O_10−δ_ (LP-LSCF, m = 0.0, 1.0, 1.5, 2.0) that displayed superior OER performance in alkaline solution. Its excellent performance can be attributed to the fact that Co^3+^ partially changed to Co^4+^ in the surface of LaSr_3_Co_1.5_Fe_1.5_O_10−δ_ and greatly generated the oxygen species O_2_^2−^/O^−^ and the O p-band center was closed to the Fermi level, which shifted the adsorption of OH^−^ and the desorption of O_2_. The noticeable OER performance gives this layered perovskite LaSr_3_Co_1.5_Fe_1.5_O_10−δ_ its promising potential for application in energy conversion and storage [54]. It was presented that the synergistic effect between two kinds of transition metal ions enhances not only the ORR and OER activity, but also the stability when Ni-doped in the LaCoO_3_ perovskites, with existing Co^3+^/Co^2+^ and Ni^3+^/Ni^2+^ changing the OH^−^ adsorption and O_2_ desorption [55]. Moreover, to improve the bifunctionality of La_0.2_S_0.8_MnO_3_ (LSM), Ni was doped into the B-sites (Figure 3A) [56]. The resulting La_0.8_Sr_0.2_Mn_0.6_Ni_0.4_O_3_ catalyst showed enhanced ORR (Figure 3B) and OER (Figure 3C) performance. The gap between OER potential (@ i = 5 mA cm^−2^) and OER potential (@ i = −1 mA cm^−2^) decreased by ~0.23 V when compared to LSM. This performance improvement in part originates from the formation of an oxygen vacancy due to the Ni doping. On the other hand, Ni doping also increased the surface area of the perovskite. Furthermore, Ni doping of LSM may also increase the conductivity of the perovskite oxide [57]. Using the e_g_ descriptor, Zhu et al. synthesized B-site substituted SrNb_0.1_Co_0.7_Fe_0.2_O_3−δ_ (SNCF) perovskite [58]. Apart from its near unity e_g_ filling, SNCF also featured small charge-transfer resistance, good OH^−^ adsorption, and oxygen desorption abilities. These beneficial features make SNCF an attractive OER catalyst over IrO_2_ and BSCF. Nb was introduced into the Mn site of CaMnO_3_ (CMO) to activate the bifunctional electrocatalysis in both OER and ORR. After being treated with H_2_, CaMn_0.75_Nb_0.25_O_3−δ_ (H_2_-CMNO) held its structure as CMO while exhibiting a reduced overpotential, a smaller Tafel slope, robust activity, and stability. Moreover, H_2_-CMNO displayed excellent ORR performance with a higher efficiency in electron-transfer when compared to CMO. This great improvement was ascribed to the stable bulk phase, appropriate e_g_ filling, and increased conductivity [59].

Non-metal element B-site doping of perovskite oxides was recently introduced to enhance the OER activity of perovskite catalysts. Zhu et al. synthesized SrCo_0.95_P_0.05_O_3−δ_ (SCP) (Figure 3D) and investigated its OER activity and electrochemical durability [60]. The potential required to generate an OER current of 10 mA cm^−2^ was only 0.48 V for SCP (Figure 3E), lower than that for its counterpart without P doping and other active perovskite catalysts. The significantly improved electric conductivity and a large number of reactive oxygen species caused by P doping were shown to be the primary cause for the improvement in activity. Moreover, the authors demonstrated that the activity of SCP increased after an accelerated durability test (Figure 3F), which was believed to be induced by the formation of an A-site deficient structure and enlarged electrochemical surface area during the test. This novel substitution strategy is important and may be used for the further rational design of perovskite electrocatalysts. Zhishan Li et al. [61] developed efficient and stable bifunctional OER and ORR catalysts in alkaline electrolyte solutions by phosphorus-doping in LaFeO_3−δ_ (LF) perovskite oxide. The enhanced catalytic activity was demonstrated to originate from the vast amount of reactive oxygen species (O_2_^2−^/O^−^ species), a trace amount of Fe^4+^ species, which could facilitate the adsorption of O_2_ and hydroxyl groups on active sites and optimal e_g_ orbital filling (t_2g_^3^e_g_^1^) as results of the P-doping. Furthermore, the stability boosted due to the incorporation of the high-valence P^5+^ cations on the Fe-site in LF.

#### 3.1.3. Perovskite Electrocatalysts with Controlled Oxygen Deficiency

**Figure 4:**
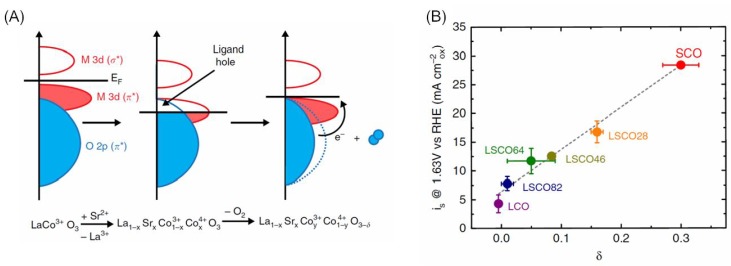
(**A**) Scheme showing the presence of oxygen vacancies with the introduction of Sr in LaCoO_3_ (adapted from [26]). (**B**) Specific OER activity of La_1−x_Sr_x_CoO_3−δ_ and IrO_2_ at 1.63 V (vs. RHE) (adapted from [26]).

The surface reactivity of perovskite oxides can be influenced by their oxygen vacancies. A RuO_2_/La_0.9_Fe_0.92_Ru_0.08_O_3_ composite by fractional Ru-substituted A-site deficient perovskite revealed a better OER activity than the pure LFRO, the conductivity of which was vastly improved through creating the deficiency [62]. One approach to introducing oxygen vacancies is to generate A-site deficiency in the oxides [63,64]. As low valence Sr was doped into the lattice of LaCoO_3_, the oxidation of Co was increased to achieve electroneutrality and the overlap of the Co 3d/O 2p band was facilitated, which further led to the generation of ligand hole/oxygen vacancy (Figure 4A) [26]. The concentration of oxygen vacancies was experimentally found to increase with the extent of the Sr doping level. To examine the effect of oxygen vacancies on its OER activity, a family of La_1−x_Sr_x_CoO_3−δ_ catalysts (LSCO, x = 0, 0.2, 0.4, 0.6, 0.8, 1.0) were synthesized. The oxygen diffusion rate of LSCO determined from chronoamperometric studies increased with Sr substitution. A good correlation between the OER activity of LSCO and its oxygen vacancy concentration/oxygen mobility was identified (Figure 4B). SrCoO_2.7_, with La fully substituted with Sr, showed the highest intrinsic OER activity, which was two times higher than that of BSCF. Oxygen vacancies can be also introduced into perovskite oxides simply through heat treatment. Chen et al. showed that oxygen-deficient BaTiO_3−x_ could be synthesized by heating the sol-gel precursor at 1300 °C under vacuum [65]. The formation of oxygen vacancies was believed to be associated with the low O_2_ partial pressure during the heat treatment. The formation of oxygen vacancies may also cause Ba deficiency due to charge compensation. The as-formed BaTiO_3−x_ showed a higher ORR and OER performance than the stoichiometric BaTiO_3_ as the oxygen vacancies could actively participate in the oxygen electrode reactions. The reductive hydrogenation process was also reported to generate oxygen-deficient perovskite oxides. For instance, CaMnO_2.5_ was synthesized by the hydrogenation of CaMnO_3_ [66]. On one hand, the Mn^3+^ in CaMnO_2.5_ has an electronic configuration of e_g_ = 1, which is favorable for OER. On the other hand, oxygen vacancies also promoted the adsorption of OH^−^ during OER. Therefore, the produced CaMnO_2.5_ showed a more enhanced OER performance over CaMnO_3_. Additionally, a functional design for S-doped CaMnO_3_ (CMO/S) nanotubes was obtained by controlled sulfur content and oxygen vacancies formed by electrospinning, heat calcination, and sulfurization treatment. The oxygen vacancies produced by S-doping contributed to the remarkable catalytic properties including excellent performance and long-term stability, which showed great potential in practical application in rechargeable metal–air batteries [67].

Bin Hua et al. reported a cation-ordered double perovskite oxide with great stability and activity in both ORR and OER, in other words, PrBa_0.85_Ca_0.15_MnFeO_5+δ_. The unique structure could not only provide ordered oxygen vacancy channels, but also furnished advisable numbers of surface oxygen defects. Additionally, the proper doping of iron maintained the B-site metals at a high valence with optimal e_g_ fillings [68].

Oxygen vacancies in perovskite thin film can be controlled by tensile strain induced by lattice mismatch between the perovskite film and the substrate [69]. Le Wang et al. figured out that compressive strain was favorable while tensile strain was unfavorable when oxygen vacancies were negligible. However, as the tensile strain increased to promote the formation of oxygen vacancies, it enabled an increase in OER [70]. A series of SrCoO_3−δ_ (0 ≤ δ ≤ 2.5) (SCO) thin films were synthesized by introducing a strain ranging from 1.0 to 4.2% [71]. Electrochemical measurements showed that the oxygen vacancies generated from the strain control were highly stable under oxidative OER conditions, which was in contrast to the previous report on the instability of SrCoO_3−δ_ prepared from the solid state synthesis [72]. SrCoO_3−δ_ perovskite thin film with a higher strain showed a higher concentration of oxygen vacancies and exhibited a higher OER activity. The optimal SCO catalyst exhibited an OER activity comparable to that of the IrO_2_ thin film. Recently, it was further shown that even a small compressive strain induced e_g_ splitting of LaNiO_3_, thus significantly enhancing its ORR and OER activity [73].

### 3.2. Perovskite Electrocatalysts with High Surface Area

Aside from its intrinsic catalytic activity, the porosity of the catalysts is also crucial for its actual performance because the porosity directly influences their active site exposure and transport of reactants/products [74,75]. The perovskite catalyst was routinely synthesized through high-temperature calcination of pre-mixed metal precursors at a solid state. Such a sintering reaction normally yields bulk perovskite with a very low surface area. A low surface area would not only limit the utilization of the active sites of the catalyst, but also restrict the wetting of the electrode by the electrolyte in battery applications. Moreover, the constrained ion transportation and electron transfer in a low surface area electrode may further decrease the battery performance of perovskite oxides. Various approaches have been explored for the preparation of perovskites with a higher surface area [76]. Sol-gel synthesis is a widely adopted method. In a typical sol-gel synthesis, a mixture solution of the precursor is first formed and the pH of the mixture solution is finely controlled to assist the reaction of the precursors. Gel products are obtained as the reaction proceeds. Calcination of the precipitants from the gel products yields the formation of the final perovskite products. Although the sol-gel derived perovskites generally show a higher surface area than that from the solid-state reaction, their particle sizes are still limited mainly to the sub-micron scale. A further decrease in the particle size is expected to boost its catalytic activity due to the enhanced active site exposure and mass transport. 

**Figure 5:**
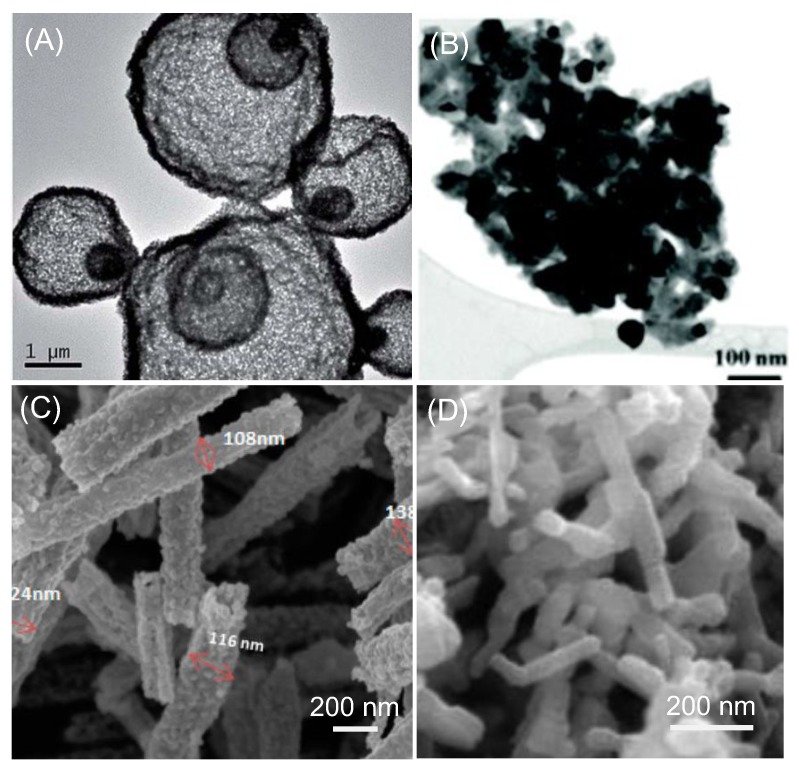
Examples of perovskite oxide electrocatalyst with a large surface area. (**A**) TEM image of multi-shelled La_0.9_Sr_0.1_CoO_3_ with a high surface area (adapted from [79], with permission from Royal Society of Chemistry, 2015). (**B**) TEM image of La-doped Ba_0.5_Sr_0.5_Co_0.8_Fe_0.2_O_3−δ_ with an average diameter of ~50 nm (adapted from [80], with permission from RSC Publishing, 2015). (**C**) SEM image of a high surface area La(Co_0.71_Ni_0.25_)_0.96_O_3__−__δ_ nanorod prepared by electrospinning (reproduced from [81], with permission from American Chemical Society, 2016). (**D**) SEM image of microporous La_0.8_Sr_0.2_MnO_3_ nanorod synthesized with a self-assembly assisted method (reproduced from [85], with permission from Elsevier, 2015).

A modified sol-gel method for the preparation of large surface area perovskite was reported by Zhuang et al. [77]. In their synthesis, activated carbon was used as porogen and pre-mixed with a citric acid solution. Corresponding metal precursors were added into the mixture solution for the formation of a gel. After drying, the gel was calcined to produce the high surface area La_0.6_Ca_0.4_CoO_3_ (LCC). A LCC catalyst with a surface area of 210 m^2^ g^−1^ was obtained. Such a huge surface area endowed the LCC with excellent ORR/OER bifunctionality. Downsizing the perovskite oxide to tens of nanometers was demonstrated with the hydrothermal assisted synthesis [78]. A hydrothermal assisted synthesis was reported to fabricate high surface area La_0.9_Sr_0.1_CoO_3_ (Figure 5A) [79]. It was shown that the morphology of the LSCO could be controlled by changing the solvent used in the hydrothermal synthesis. When a hybrid solvent composed of ethanol and water was used, multi-shelled LSCO nanoparticles with a surface area up to 23.82 m^2^ g^−1^ were prepared. In contrast, the LSCO nanoparticles prepared with the conventional sol-gel method had a surface area of only 11.65 m^2^ g^−1^. 

Jung et al. demonstrated that doping and heat treatment could be used to enhance the surface area of the perovskite in the case of La-doped Ba_0.5_Sr_0.5_Co_0.8_Fe_0.2_O_3−δ_ (BSCF) perovskite (Figure 5B) [80]. The calcination temperature influenced the phase of the resulting perovskite and a uniform cubic perovskite phase could be formed in a certain temperature range. It was also suggested that particle growth was suppressed at a higher La doping level because the diffusion required for ion migration was inhibited. La_0.7_BSCF synthesized at 700 °C exhibited a BET area of 21.3 m^2^ g^−1^. Due to the combination of a high surface area and unique electronic structure, La_0.7_BSCF possessed excellent ORR and OER activity and a superior Zn–air battery performance compared to the 20% Pt/C catalyst. To increase the surface area of a perovskite catalyst, the electrospun technique was used (Figure 5C) [81,82,83]. Highly porous La_0.5_Sr_0.5_Co_0.8_Fe_0.2_O_3_ (LSCF) nanorods with a surface area of 36.5 m^2^ g^−1^ have been reported [82]. When coupled with nitrogen-doped graphene, the LSCF nanorod exhibited a comparable ORR performance and better OER performance when compared to the commercial Pt/C catalyst. The superior stability of the composite was also demonstrated. Hierarchically structured La_0.5_Sr_0.5_CoO_3−x_ with both mesopores and macropores were also synthesized and used for a Li–O_2_ battery [83]. Dai et al. reported perovskite oxides LaFeO_3_ (LF) through building 3D ordered macroporous (3DOM) architecture in combination with bulk lattice doping. The open and interconnected 3DOM structure led to a high specific area (20 m^2^ g^−1^), which also increased the number of active sites and facilitated mass transfer. A small amount of iron substitution with cobalt in the oxide lattice (3DOM-LaFe_0.8_Co_0.2_O_3_/LFC82) further promoted the catalytic ability and durability in OER [84].

Lu et al. presented a self-assembly method to prepare La_0.8_Sr_0.2_MnO_3_ (LSM) nanorods with numerous defects and high surface area (20.6 m^2^ g^−1^) by using cetrimonium bromide (CTAB) as the soft template (Figure 5D) [85]. Benefiting from the high porosity, the LSM nanorods showed improved electrocatalytic activity toward both ORR and OER compared to the LSM nanoparticles derived from the sol-gel method. Yang et al. reported a graphene/meso-LaSrMnO composite using a surfactant as the structure-directing agent [86]. This novel composite contains both mesopores (in LaSrMnO) and macropores (between the nanosheets) and thus allows for efficient ion transportation and ion diffusion during battery operation. In addition, the composite is more mechanically robust when compared to the traditional electrode based on perovskite nanoparticles. The Li–O_2_ battery assembled with the graphene/meso-LaSrMnO composite showed both high discharge capacity and cycling performance. 

Recently, it was found that changing the porosity of perovskite oxides may alert its electronic state. In the case of LiCoO_2_ (LCO), a high-spin state (t_2g_^4^e_g_^2^) is more favorable for cobalt ions at the surface of nano-sized LCO [87]. As the size of the LCO nanoparticle decreases, the number of surface cobalt ions increases and the e_g_ filling increases as a result. As derived from the magnetization measurements, the e_g_ was 1.0, 1.1, 1.2, and 1.27 for bulk LCO, 200 nm LCO, 80 nm LCO, and 60 nm LCO, respectively. To investigate the intrinsic OER activity of the LCO of different sizes, the OER reaction currents of the samples were normalized to their BET surface area. It was found that 80 nm LCO exhibited the highest activity. The 60 nm LCO had a higher charge-transfer resistance because of excessive e_g_ filling. Therefore, the OER activity of 60 nm LCO was inferior to that of the 80 nm LCO. This observation is in good agreement with the Suntivich principle [17], where an e_g_ filling of ~1.2 is optimal for electrochemical OER. The results of this study demonstrate that a small particle size is not always favorable for perovskite catalysts and multiple factors should be considered when designing perovskite catalysts in the nanoscale. 

### 3.3. Composite Electrocatalysts Based on Perovskites

**Figure 6:**
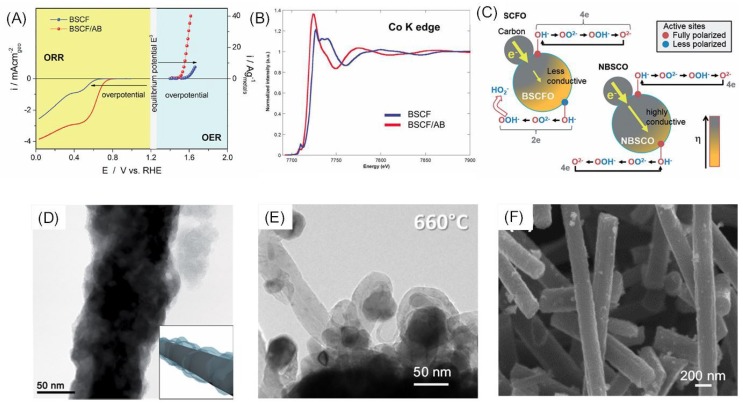
(**A**) Overall ORR and OER performance of Ba_0.5_Sr_0.5_Co_0.8_Fe_0.2_O_3−δ_ (BSCF) and BSCF/acetylene black (**A**,**B**) composite in O_2_ saturated 0.1 M KOH solution at a rotating speed of 1600 rpm with a scanning speed of 5 mV s^−1^ (adapted from [93], with permission from John Wiley and Sons, 2015). (**B**) XANES spectra at the Co K edge for BSCF and BSCF/AB composite (adapted from [93], with permission from John Wiley and Sons, 2015). (**C**) Proposed ORR mechanism on a carbon supported perovskite oxide electrocatalyst (adapted from [96], with permission from John Wiley and Sons, 2015). (**D**) TEM image of the cloud-like graphene nanoplatelet functionalized Nd_0.5_Sr_0.5_CoO_3−δ_ nanorod electrocatalyst (adapted from [97]) (**E**) TEM image of the CVD grown nitrogen doped carbon coated La_0.58_Sr_0.4_Fe_0.2_Co_0.8_O_3_ electrocatalyst (adapted from [98], with permission from Elsevier, 2016). (**F**) SEM image of the nitrogen doped carbon nanotube embedded with LaTi_0.65_Fe_0.35_O_3−δ_ (adapted from [99], with permission from Elsevier, 2015).

The combination of perovskite oxides with another component that is either active in ORR/OER or provides conductive pathways is an effective way to improve the performance of the perovskite catalysts. Nanocarbons are the most commonly used composite with perovskite oxides. It has generally been thought that the role of carbon in perovskite/carbon composites is only to provide conductive pathways in oxygen electrode reactions [88,89]. The electrocatalytic performance for the ORR of carbon-supported La_0.5_Sr_0.5_MnO_3_ perovskite was obviously promoted [90]. A three-component-integrated catalyst (i.e., LaSrMnO/Fe_3_C/Carbon) such as La_0.45_Sr_0.45_Mn_0.9_Fe_0.1_O_3−δ_ could be fabricated by a facile surface chemistry approach, which is a feasible bifunctional catalyst for ORR and OER [91]. Additionally, there appeared to be a synergistic effect between the perovskite-oxide and the XC-72, with a change from the two-step, 2e^−^ pathway to the 4e^−^ transfer pathway as the carbon content increased [92]. Using Ba_0.5_Sr_0.5_Co_0.8_Fe_0.2_O_3−δ_/acetylene black carbon (BSCF/AB) as the model catalyst, Fabbri et al. proposed that there existed interactions between the carbon support and the oxide (Figure 6A) [93]. The Co K edge peak of BSCF/AB in the x-ray absorption near-edge structure (XANES) spectroscopy shifted toward the lower energy region compared to that of the pristine BSCF (Figure 6B), indicative of a lower oxidation of Co in BSCF/AB than in BSCF. The existence of a lower Co oxidation state in BSCF/AB was further supported by the XRD measurements, which showed that the BSCF/AB had a slightly higher lattice parameter. The presence of the lower Co oxidation state in BSCF/AB was attributed to the reduction of Co by carbon, which is thermodynamically favorable because Co has a high density of state near the Fermi level. The presence of lower valence Co helps to increase the intrinsic conductivity of the BSCF, thus allowing for both enhanced ORR and OER activity [94]. Additionally, carbon may effectively catalyze the disproportionation of HO_2_^−^ to generate O_2_, which might be the rate-determining step for the perovskite catalyzed ORR. Therefore, a pseudo-four-electron ORR pathway could be established on the carbon-supported perovskite [25,95]. For OER, carbon support facilitates the disproportionation of the intermediate peroxide [25]. Due to this effect, carbon support can enhance the OER activity of perovskite with limited HOO^−^ disproportionation capability. In addition, heteroatoms in the carbon support may contribute to the stabilization of the perovskite catalyst during oxidative conditions of OER. Recently, a model describing the ORR mechanism of carbon supported perovskite catalysts was proposed [96] and suggested that, when the electrical conductivity of the perovskite phase was low, there was a polarization gradient in the catalyst at a given overpotential (Figure 6C). ORR occurred at the highly polarized region (phase boundaries where carbon and perovskite make contact) and proceeded through a four-electron pathway while the ORR at low polarization sites (electrolyte–perovskite interface) underwent a two-electron pathway. With the increase of the overpotential or addition of carbon contents, the polarization degree of the originally low polarization sites increased and the overall electron transfer number approached ~4. It was also suggested that adding conductive carbon contents into perovskite with low conductivity may decrease the number of rate-determining steps in ORR.

Different carbon nanostructures are employed for the construction of high-performance perovskite composite catalysts. Edge-iodinated graphene nano-pellets (IGnP) were synthesized and coated onto Nd_0.5_Sr_0.5_CoO_3−δ_ (NSC) nanorods by sonication (Figure 6D) [97]. IGnP was used because of its intrinsic ORR activity, while NSC was chosen as the OER active component for its intermediate e_g_ value (~1). IGnP enhances the affinity of O_2_ on the composite and functions as active catalytic sites. Apart from its good ORR activity, the IGnP/NSC composite also showed an enhanced OER performance when compared to the IrO_2_ catalyst. Carbon nanotubes have also been used to enhance the bifunctionality of perovskite oxides. The nitrogen-doped carbon nanotubes (NCNTs) were grown on La_0.58_Sr_0.4_Fe_0.2_Co_0.8_O_3_ (LSFC) to impart LSFC with high ORR activity (Figure 6E) [98]. Prabu et al. used an electrospinning method to produce LaTi_0.65_Fe_0.35_O_3−δ_ (LTF)/nitrogen-doped carbon nanorods (Figure 6F) [99]. The nanorod exhibited a compact structure with LTF homogeneously embedded within the carbon skeleton. The LTF/N-doped carbon nanorod showed a high ORR activity through a four-electron pathway. In contrast, the LTF nanorod with a similar morphology showed a two-electron selectivity in ORR. In addition, the composite LTF demonstrated a higher OER performance as well as durability. These results clearly prove the synergistic effect between the LTF and nitrogen-doped carbon phase in the ORR and OER process. Dong-Gyu Lee et al. presented simply mixed, composite catalysts of perovskite oxide catalysts and polypyrrole (pPy). The mixture of catalyst particles and pPy/C significantly reduced the overpotential of the ORR and OER [100]. Miguel A. reported dual-doped graphene/perovskite mixtures combining boron, nitrogen, phosphorous, and sulfur precursors were co-reduced with graphene oxide and mixed with La_0.8_Sr_0.2_MnO_3_ (LSM), which increased their bifunctional catalytic activity [101]. Due to the high intrinsic activity of the NCNTs, the composite exhibited a pronounced increase in ORR activity. A NCNT-wrapped LaNiO_3_ bifunctional catalyst was also developed [102]. It was believed that the NCNT framework not only provided an efficient electron transport pathway, but also facilitated the mass transfer at the electrode [102]. 

Combinations of ORR-active La_0.8_Sr_0.2_MnO_3−δ_ (LSM) and OER-active Ba_0.5_Sr_0.5_Co_0.8_Fe_0.2_O_3−δ_ (BSCF) have also been reported [103]. To exclude the effect from surface area and additives, a thin film of BSCF/LSM composite was formed on a Nb-doped SrTiO_3_ (NSTO) substrate by pulsed laser deposition. The BSCF coverage on LSM was varied to investigate the effect of the film composition on the overall catalytic activity toward ORR and OER. As the BSCF coverage increased, the overall ORR and OER activity of the catalyst was enhanced. The sum of the ORR and OER overpotential was as low as 0.7 V when the surface coverage of BSCF approached 94%. Furthermore, the BSCF decoration was shown to promote the stability of the film under working conditions. Metal oxides were also compounded with perovskites to enhance the activity of perovskite catalysts. A MnO_2_-LaNiO_3_ catalyst was reported to show enhanced ORR and OER activity when compared to both MnO_2_ and LaNiO_3_ [104]. The detailed mechanism for this enhancement is not clear yet. To improve the OER property of La_1−x_Sr_x_MnO_3_ (LSM), NiCo_2_O_4_ was homogeneously dispersed on a LSM nanorod. The combination of the ORR-active LSM and OER-active NiCo_2_O_4_ led to the formation of an efficient bifunctionality that showed excellent rate capability as well as cycle stability in the Li–O_2_ battery. Daehee Lee et al. demonstrated an improvement of ORR activity in (La_0.8_Sr_0.2_)_0.95_MnO_3_ perovskite thin-films placed on different oxide supports. XAS and EIS were performed to confirm that the atomic orbital interactions between the support and the catalysts sped up the exchange kinetics, thus improving the ORR electrocatalytic capability [105]. Meng Li et al. took the advantages of the junction RuO_2_/perovskite with charge transfer dynamics, which enabled excellent OER activity in the solid oxide electrolysis cell (SOEC) at lower temperatures [106].

## 4. Conclusions and Discussion

The ORR and OER process are rate-determining reactions in many energy conversion processes. Thus, the development of efficient, low-cost, and bifunctional electrocatalysts is essential for the advancement of these technologies. Recently, perovskite oxides have been demonstrated as promising ORR/OER bifunctional catalysts. In addition, the performance of perovskite electrocatalysts may be further improved due to the high flexibility to tune the electronic structure of the oxides. In this review, we summarized the most recent progress on the development of high-performance perovskite oxygen electrocatalysts. As reviewed above, achievements have been accomplished in developing highly active perovskite oxide catalysts for ORR/OER. However, corresponding studies are still in their early stage and there are still many challenges to meet to apply perovskite catalysts at a large scale. Some of the issues to be addressed are as follows. First, despite the efforts to understand the ORR/OER mechanism on perovskite catalysts, it is still challenging to design and optimize perovskite catalysts rationally due to the complexity of the catalytic process. In addition, the actual performance of perovskite catalysts is influenced by multiple interplaying intrinsic factors such as electronic state and porous structure. To better clarify the ORR/OER mechanism of the perovskite oxides, well-defined model perovskites should be developed to correlate the bulk electronic structure and surface chemistry of perovskite oxides with their intrinsic electrocatalytic performances. Second, while partial substitution of A/B-site cations of perovskite oxides may enhance its activity, it may also deteriorate the stability of the perovskite. To guide the future development of partially substituted perovskite oxides, further studies are required to illustrate the relationship between the stability and activity of the perovskites. For composite perovskite electrocatalysts, the synergy between the oxide and the secondary component is not fully understood yet. Studies in this direction may help to fully utilize the synergistic effect to maximize the activity of the composite perovskite catalysts. Third, the ORR/OER catalytic activity of the perovskites should be further improved. Tuning the composition and surface area or using appropriate support materials have been demonstrated as effective methods to enhance the activity of perovskite oxides. Combining these methods in the development of perovskite catalysts may further boost the electrocatalytic performance. Fourth, although perovskite oxides have shown relatively high stability, it was observed in many studies that the oxides underwent structural changes during catalysis. Strategies that could inhibit this structural change would be useful for the practical applications of perovskite electrocatalysts. Finally, despite the feasibility of tuning the perovskite electrocatalyst through morphology engineering or controlling its composition, the perovskite should be designed carefully because the perovskite structure could possibly be broken down due to the stress generated during the morphology/composition control. Thus, the catalytic performance and stability of perovskite oxide should be well balanced in the design of the oxygen electrocatalyst. Although some key challenges still need to be addressed, perovskite oxygen electrocatalysts are highly promising for advanced energy conversion devices such as metal–air batteries. With the remarkable progress achieved recently and on-going research efforts, widespread applications of perovskite oxygen electrocatalysts can be expected in the near future.

## Abbreviations

ORR	oxygen reduction reaction
OER	oxygen evolution reaction
RHE	reversible hydrogen electrode
TEM	transient electromagnetic method
SEM	scanning electron microscope
DFT	Density Functional Theory
XANES	X-ray absorption near-edge structure spectroscopy
XRD	X-ray diffraction
CVD	Chemical Vapor Deposition
BET	initials of Brunauer, Emmett and Teller
CTAB	cetrimonium bromide
BSCF5582	Ba_0.5_Sr_0.5_Co_0.8_Fe_0.2_O_3-δ_
CYM	Ca_0.9_Yb_0.1_MnO_3_
LSCF	LaSr_3_Co_m_Fe_3-m_O_10-δ_
SNCF	SrNb_0.1_Co_0.7_Fe_0.2_O_3-δ_
CMO	CaMnO_3_
CMNO	CaMn_0.75_Nb_0.25_O_3-δ_
SCP	SrCo_0.95_P_0.05_O_3-δ_
LF	LaFeO_3-δ_
LFRO	La_0.9_Fe_0.92_Ru_0.08_O_3_
LSCO	La_1-x_Sr_x_CoO_3-δ_
SCO	SrCoO_3-δ_
LCC	La_0.6_Ca_0.4_CoO_3_
LCO	LiCoO_2_
LSM	La_0.8_Sr_0.2_MnO_3_
IGnP	Edge-iodinated graphene nano-pellets
NSC	Nd_0.5_Sr_0.5_CoO_3-δ_
NCNTs	nitrogen-doped carbon nanotubes
NSTO	Nb-doped SrTiO_3_
SOEC	solid oxide electrolysis cell
